# Bullous immunoglobulin A dermatosis following oral contrast in a patient with dermatitis herpetiformis

**DOI:** 10.1016/j.jdcr.2026.04.047

**Published:** 2026-04-30

**Authors:** Nikolas G. Hernandez, Jamie L. Karch, David Grand, Alicia T. Dagrosa

**Affiliations:** a Dartmouth Geisel School of Medicine, Hanover, New Hampshire; b Department of Dermatology, Dartmouth-Hitchcock Medical Center, Lebanon, New Hampshire; c Department of Pathology and Laboratory Medicine, Dartmouth-Hitchcock Medical Center, Lebanon, New Hampshire

**Keywords:** autoimmune subepidermal blistering, contrast media, dermatitis herpetiformis, IgA-mediated blistering, linear IgA bullous dermatosis, oral contrast-induced reaction

## Introduction

Linear IgA bullous dermatosis (LABD) and dermatitis herpetiformis (DH) are rare IgA-mediated subepidermal blistering disorders with overlapping histology but distinct immunopathology and clinical patterns. We report a unique case of acute bullous IgA-mediated dermatosis occurring shortly after oral contrast administration. Oral contrast agents have not previously been reported as triggers of LABD, with only a single prior case linked to DH. This case highlights the diagnostic challenges in distinguishing LABD from DH in such cases.

## Case report

A 66-year-old woman with a history of celiac disease, dermatitis herpetiformis (DH) managed with dapsone, dermatomyositis, protein C deficiency, peripheral artery disease, and prior venous thromboembolism on apixaban presented to the emergency department with a 4-day history of painful, pruritic blistering rash accompanied by nausea and vomiting. One day before rash onset, she received oral and intravenous iodinated contrast (iohexol and iodixanol) during gastric cancer evaluation prompted by a positive anti-TIF1-gamma antibody. She reported strict adherence to a gluten-free diet, although celiac serologies were not obtained during this admission, and had remained on stable dapsone therapy for dermatitis herpetiformis, with no recent medication changes aside from a gabapentin to pregabalin switch weeks prior and a warfarin to apixaban transition 1 month prior. Initially treated at an external emergency department with steroids for presumed erythema multiforme, her rash worsened with new blistering involvement including the oral, nasal, and genital mucosa, along with hoarseness and dysuria. Due to concerns about Stevens-Johnson syndrome/toxic epidermal necrolysis, she was referred to our center.

**Fig 1:**
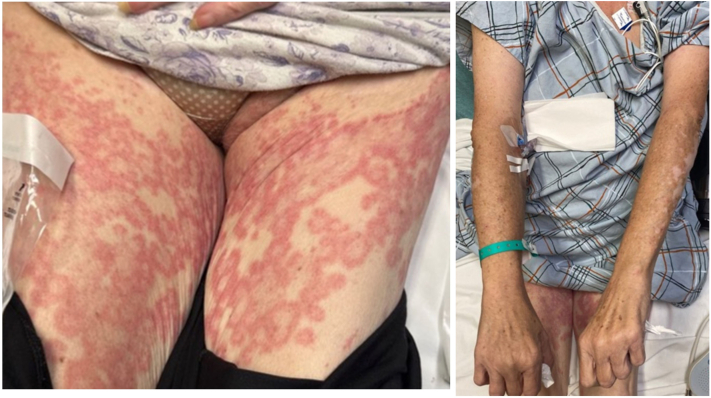
Polycyclic, edematous *pink* to *light-brown* annular plaques on the forearm and trunk at initial presentation in the emergency department.

**Fig 2:**
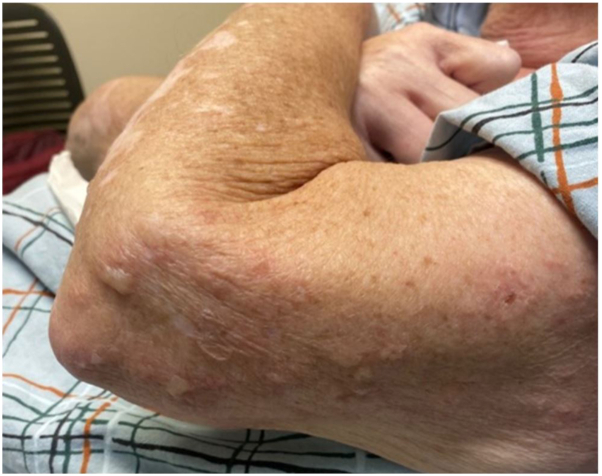
Progression of rash several hours later with development of tense vesicles and bullae.

Upon initial presentation to our emergency department, the patient exhibited polycyclic, edematous pink-light brown plaques on the bilateral forearms, thighs, back, and abdomen, sparing the palms and soles (Fig 1). Over several hours, these plaques evolved into tense vesicles and bullae on an erythematous base, affecting the face (including nares), ears, and upper extremities (Fig 2). Faint purplish macules appeared on the bilateral palms, with no evidence of conjunctival or mucosal erosions or crusting. Nikolsky sign was negative. Vital signs were stable without fever or signs of infection. A punch biopsy was performed, and the patient was placed on precautions for disseminated herpes simplex virus and initiated on prophylactic acyclovir. Topical treatments included triamcinolone 0.1% cream and hydrocortisone 2.5% ointment for the face, with as-needed hydroxyzine for itch.

**Fig 3:**
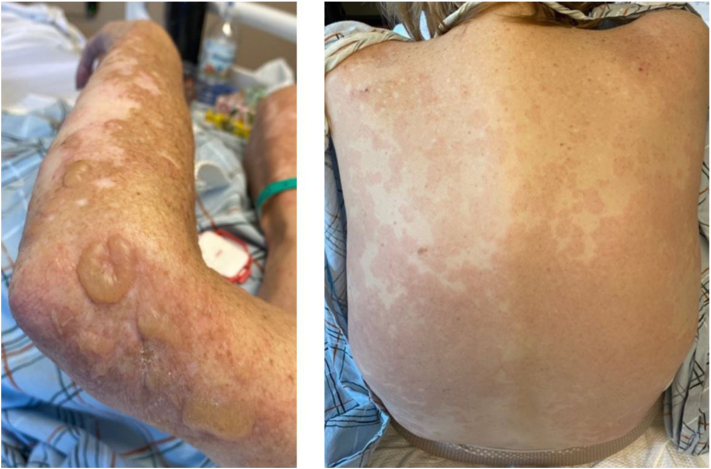
Bullous progression on the following day on the bilateral upper extremities (left), with resolving rash and residual postinflammatory hyperpigmentation (right).

**Fig 4:**
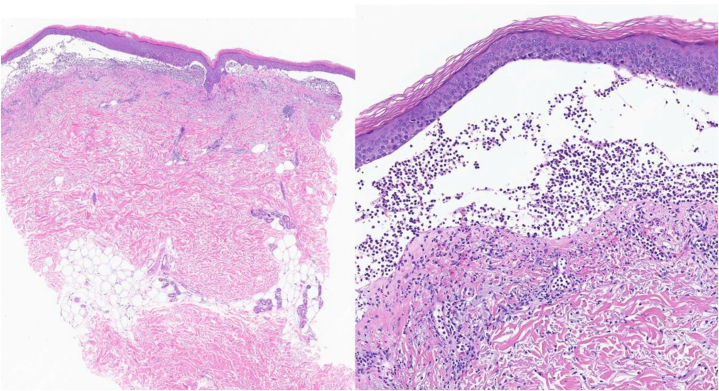
Bullous IgA dermatosis. H&E, 4× (left) and 30× (right), demonstrating a subepidermal blister with associated inflammatory infiltrate and neutrophil-predominant infiltrate.

The following day, bullae became more prominent on the upper extremities while erythema diminished, leaving residual postinflammatory hyperpigmentation (Fig 3). In response to this, the patient’s dapsone dose was increased from 50 mg twice daily to 75 mg twice daily. HSV polymerase chain reaction and cultures returned negative, and acyclovir was discontinued. Biopsy findings revealed a subepidermal blister with a dense neutrophil-rich perivascular and interstitial inflammatory infiltrate (Fig 4). Papillary neutrophilic microabscesses were absent. Direct immunofluorescence (DIF) showed strong granular IgA deposition along the dermal-epidermal junction without accentuation along the dermal papillae.

Over the subsequent days, the bullae continued to diminish in size, and the rash resolved with residual postinflammatory hyperpigmentation. The patient was discharged without subsequent worsening of the rash. The patient was subsequently lost to follow up.

## Discussion

Differentiating LABD from DH can be challenging, as both are IgA-mediated autoimmune blistering diseases with overlapping clinical, histologic, and immunopathologic features. Accurate diagnosis requires integration of clinical morphology and distribution with histopathologic and direct immunofluorescence findings rather than reliance on a single criterion.

In LABD, disease may be idiopathic or drug-induced and is most commonly triggered by vancomycin.^1^ Clinically, it is characterized by the acute onset of tense vesicles and bullae that often form annular or “cluster of jewels” configurations, frequently involving the trunk and face, with mucosal involvement reported in up to 50% of cases. Histopathology typically demonstrates a subepidermal blister with a neutrophil-predominant inflammatory infiltrate, and DIF most often reveals IgA deposition along the basement membrane zone.

In contrast, DH is strongly associated with gluten-sensitive enteropathy and classically presents as intensely pruritic, grouped vesicles distributed symmetrically on extensor surfaces.^2^ Mucosal involvement is rare. Histopathology typically demonstrates neutrophilic microabscesses within the dermal papillae, and DIF characteristically reveals granular “picket-fence” IgA deposition within the papillary dermis.^3^

Despite the patient’s established history of DH, her acute presentation was more consistent with LABD. The rapid onset of widespread annular tense bullae, involvement of the face and trunk, and reported mucosal symptoms favored LABD over classic DH. Although pruritus is a hallmark feature of DH, it is not exclusive to that diagnosis and may also be present in LABD.

Histologic findings in this case supported LABD, demonstrating a subepidermal blister with a dense neutrophil-rich infiltrate. Diagnosis can be challenging in nonclassic DIF patterns, particularly when IgA is not strictly linear or confined to dermal papillae. While LABD typically shows linear IgA along the basement membrane zone, a subset demonstrates granular or papillary accentuation. Conversely, DH typically shows granular IgA in dermal papillae but may rarely extend along the basement membrane zone. In this case, granular IgA along the dermal-epidermal junction without papillary accentuation represents an uncommon overlapping pattern.^4^^,^^5^ Correlation with the overall clinicopathologic findings favored LABD.

Although oral contrast has not previously been reported as a trigger for LABD and has been linked to DH in only a single prior case report,^6^ the close temporal relationship between exposure and symptom onset, absence of alternative triggers, and strict adherence to a gluten-free diet suggest oral contrast as the likely precipitating factor for LABD in this patient. Pregabalin has not been convincingly associated with LABD or DH, and reported cutaneous reactions are typically nonspecific, making it a less likely trigger in this case.

This case identifies oral contrast as a potential novel trigger for LABD and underscores the diagnostic challenges posed by IgA-mediated blistering disorders, highlighting the importance of clinicopathologic correlation when distinguishing LABD from DH.

## Conflicts of interest

None disclosed.
